# Clozapine-Induced Constipation: A Case Report and Review of Current Management Guidelines

**DOI:** 10.7759/cureus.14846

**Published:** 2021-05-04

**Authors:** Rikinkumar S Patel, Nikhila Veluri, Alex Suchorab, Kaushal Shah, Geetika Verma

**Affiliations:** 1 Psychiatry and Behavioral Sciences, Oklahoma State University, Tulsa, USA; 2 Psychiatry, Griffin Memorial Hospital, Norman, USA; 3 General Medicine, American University of Integrative Sciences, St. Michael, BRB; 4 General Medicine, Medical University of the Americas, Charlestown, KNA

**Keywords:** treatment-resistant schizophrenia, clozapine-induced constipation, schizophrenia spectrum disorders, fecal impaction, colonic hypomotility

## Abstract

Clozapine is a second-generation antipsychotic recommended after the failure of two or more antipsychotics for treatment-resistant schizophrenia. Clozapine proved to also decrease recurrent suicidal behaviors in schizophrenia spectrum disorders. Yet, physicians often use clozapine as a last resort despite its proven efficacy due to its side effect profile. A noted side effect of clozapine is agranulocytosis, which requires a weekly complete blood count with differentials. Clozapine's anticholinergic activity causes colonic hypomotility, leading to constipation, and only a few studies examined clozapine-induced constipation (CIC). Few of the reported complications of CIC include bowel obstruction or bowel perforation due to fecal impaction. Herein we document a case report of CIC and also conducted a review of published case reports examining the complexity and management of CIC. CIC is a critical condition if unresolved as it can lead to mortality. Future directions and guidelines should be developed for early diagnosis and treatment for CIC, which will provide reassurance and directions for both the physicians and patients.

## Introduction

Clozapine is a second-generation antipsychotic (SGA) approved by the food and drug administration (FDA) for treatment-resistant schizophrenia. Clozapine is also used for recurrent suicidal behaviors in schizophrenia and schizoaffective disorders. Clozapine is preferably started after an insufficient response with at least two prior antipsychotics [1].

Antipsychotic effects of clozapine are exerted due to antagonism at both serotonin receptor (5-HT2A) receptors and dopamine receptors (D1, D2, D3, and D5), with a high affinity for the dopamine D4 receptor. Antagonism of the serotonin receptor 5-HT2A receptor helps with the improvement of depression and negative cognitive symptoms, and antagonism of dopamine receptors reduces the positive symptoms associated with schizophrenia. In addition, clozapine exerts limited antagonistic activity on adrenergic, cholinergic, and histaminergic receptors, thereby accounting for potential side effects including constipation, urinary incontinence, and excessive salivation [2].

A well-known adverse effect of clozapine is agranulocytosis, requiring mandatory close monitoring of absolute neutrophil count. Cardiomyopathy or myocarditis, metabolic syndrome, seizures, and gastrointestinal (GI) hypomotility are other side effects of clozapine. Clozapine-induced constipation (CIC) due to GI hypomotility affects up to 80% of users but is less frequently explored [3].

Colonic motility occurs through haustral contraction and mass movement. Neurotransmitters, such as serotonin, acetylcholine, and nitric oxide, are released during distension of the gut wall, colon muscle contraction, and relaxation that aid in propelling the bolus forward. A disruption that slows colonic transit time (colonic hypomotility) induces constipation. Many antipsychotics have anticholinergic properties; however, the number of reported CIC cases and its severe effects are increasing [4]. CIC’s risk rises with higher dosages and leads to severe complications like peritonitis, fecal impaction, aspiration of feculent emesis, bowel obstruction, bowel perforation, adyanamic ileus, and colonic ischemia [5].

In this case report, we present a case of CIC and discuss the clinical presentation, complications, prognosis, and management of CIC.

## Case presentation

The patient was an 18-year-old Caucasian female diagnosed with schizoaffective disorder, intermittent explosive disorder (IED), intellectual development disorder (IDD), and attention deficit hyperactivity disorder (ADHD). She has a longstanding history of psychiatric inpatient hospitalizations for suicidal and non-suicidal self-injurious behavior, mood lability, and aggression. Upon admission, her medications were olanzapine 10mg three times daily (TID) for psychosis, valproate extended-release (ER) 500mg TID for mood, and lorazepam 0.5mg TID for aggression. Clozapine 25mg twice daily (BID) was initiated due to the patient’s history of failed trials with first-generation antipsychotics (FGA) and SGA. This dosage of clozapine was titrated up to 150mg BID over seven days in increments of 25mg. Two longer-acting injectables (LAIs) antipsychotics in intramuscular (IM) formulation were added for worsening psychosis. So, the patient was on three antipsychotics for treatment-resistant schizophrenia: clozapine 150mg BID, haloperidol decanoate 200mg IM every four weeks, and paliperidone 234mg IM every four weeks.

On day 22 of clozapine use, the patient developed symptoms of constipation. As per medical consult, polyethylene glycol 17mg BID and prune juice with meals daily were added and did not improve her symptoms. Nursing reports indicated that the patient had adequate food and fluid intake; however, she continued to complain of abdominal pain and bloating. The patient was then started on bisacodyl 1mg daily, without sufficient relief. On day 36, her abdominal x-ray revealed stool throughout her colon without any bowel distension or obstruction. CIC was suspected as the patient had a total score of 5 on the Naranjo Adverse Drug Reaction Probability Scale [6]. She was then managed with two doses of magnesium citrate enema 150ml, allowing her to have normal bowel transit within six hours. No complications, in addition to constipation, were noted from clozapine treatment in our patient.

## Discussion

Schizophrenia patients exhibit increased pain tolerance and threshold [7]. The inverse relationship between increased severity of the psychotic symptoms and decreased pain sensitivity is concerning. Patients experiencing severe psychosis may be unable to express their perceived pain and medication side effects. Additionally, anticonvulsant mood stabilizers and antipsychotic medications may also alter pain sensitivity. Moreover, the negative symptoms of schizophrenia (flat affect and apathy) may affect the facial expression of pain, leading physicians to perceive the symptoms as mild [7].

Clozapine dosage in these patients ranged from 100mg to 900mg daily and was used from two weeks to 20 years. As per current guidelines, effective management of treatment-resistant schizophrenia recommends a clozapine level of 350 nanograms per milliliter (ng/ml) [8]. A higher dosage of clozapine is a risk factor for developing CIC [9]. Patients develop common clinical symptoms of severe diffuse abdominal pain and constipation with clozapine, although the duration of time for receiving clozapine treatment and the onset of symptoms varied substantially. Clozapine-induced agranulocytosis has the highest risk of occurrence in the first two months; however, CIC can occur at any time during the treatment course. Constipation is a common side effect seen with clozapine and occurs regardless of treatment duration [2]. CIC is under-recognized, and the severity of symptoms may be nonspecific or mild. Other symptoms experienced by these patients included abdominal distention, nausea, vomiting, tachycardia, hypotension, hypothermia, dehydration, and altered mental status.

Diagnostic tests included routine blood work, and imaging tests included an abdominal x-ray, computerized tomography (CT) scan, and abdominal ultrasound. The management of CIC was dependent on symptom severity. For mild symptoms, laxatives agents like psyllium, polyethylene glycol, prochlorperazine, and enemas were used [10-12]. Moreover, for cases with a lack of symptom improvement, further diagnostic imaging was done, followed by interventions including urgent laparotomy, manual decompression, and surgery (i.e., colectomies and ileostomies). Nasogastric suction and intraperitoneal drainage had been used in patients presenting with vomiting or perforation of the colon, respectively.

Complications in the published cases were bowel obstruction, septic shock, peritonitis, pneumonia, bowel infarction/perforation, and abdominal compartment syndrome [8,10,12-18]. Death was observed in patients on a higher clozapine dosage. The cause of death in 50% of these cases was due to sepsis, either from infection or post-surgical complication [5,8,14,15]. Other complications that lead to death were bowel infarction, bowel obstruction, abdominal compartment syndrome, and severe uncontrollable diarrhea. Patients were reinitiated or stayed on clozapine without any severe consequences of CIC [10,12,13,16,18-20]. However, these patients were prescribed daily laxatives with clozapine. Rege and Lafferty reported in their case report that restarting clozapine post-manual disimpaction and laparotomy instigated another episode of CIC with fecal impaction. This patient required switching clozapine to risperidone 2mg daily with weekly fleet enema that provided medical stabilization [21].

Before starting clozapine, a detailed medical history with risk factors for constipation must be obtained. Physical examination and dietary consult should be a part of the pre-treatment protocol [8]. So, the clinicians should provide psychoeducation, including awareness of side effects like constipation, and physicians should screen the patients for most common symptoms like constipation to prevent CIC. CIC can be prevented by asking the patients to maintain adequate hydration, increasing dietary fiber intake, and obtaining regular exercise [15,21]. Early diagnosis of CIC significantly reduces the incidence of invasive interventions and future complications in schizophrenia patients, thereby reducing the mortality rate by about 50% [9].

Patients on clozapine are managed with lubiprostone to prevent chronic constipation [10]. Lubiprostone is a prostaglandin E1 analog that activates the chloride channels on the gastrointestinal epithelial cells’ luminal surface. It softens the stool by increasing luminal secretions rich in chloride. Despite this benefit, lubiprostone use for chronic constipation is very low. In one of the treatment-resistant schizophrenia patients with a history of clozapine-induced ileus and small bowel obstruction, clozapine management was continued and supplemented with docusate, lactulose, and lubiprostone with no other adverse events like CIC [10]. Tapering down the clozapine dose is the first‐choice treatment strategy with or without changing to antipsychotic with less anticholinergic effects. Levin et al. found a 25% reduction in clozapine dosage by substituting it with quetiapine 2mg for every 1mg of clozapine. This combination regimen effectively reduced weight and improved glycemic control in patients who were previously only on clozapine [8].

## Conclusions

Future directions and guidelines should be developed for early diagnosis and treatment for CIC as it is a critical condition if unresolved leading to mortality. The current management strategies for CIC include adequate hydration, osmotic agents (e.g., lactulose or polyethylene glycol), stimulant laxatives (e.g., senna or bisacodyl), and enema. The use of bulk‐forming or fiber‐based laxatives are not recommended since CIC’s pathophysiology is due to GI hypomotility. Clinicians should consider lubiprostone in chronic and untreatable constipation because of its stool softening properties. Patients managed with clozapine have greater severity of illness and morbidity that may require chemical restraints, and in such situations, clinicians should prefer SGA with none/minimal anticholinergic activity like ziprasidone, risperidone, and aripiprazole.
